# Activity disruption, illness intrusiveness, and life satisfaction in adults with type 1 diabetes: an ecological momentary assessment study

**DOI:** 10.1007/s10865-026-00643-9

**Published:** 2026-03-07

**Authors:** Yujia Mo, Raymond Hernandez, Stefan Schneider, Jeffrey S. Gonzalez, Claire J. Hoogendoorn, Valerie Tapia, Elizabeth Pyatak

**Affiliations:** 1 Chan Division of Occupational Science and Occupational Therapy, University of Southern California, Los Angeles, CA USA; 2 Center for Economic and Social Research, University of Southern California, Los Angeles, CA USA; 3 Department of Psychology, University of Southern California, Los Angeles, CA USA; 4 Leonard Davis School of Gerontology, University of Southern California, Los Angeles, CA USA; 5 Division of Endocrinology and Diabetes, Department of Medicine, Albert Einstein College of Medicine, Bronx, NY USA; 6 Department of Epidemiology and Population Health, Albert Einstein College of Medicine, Bronx, NY USA; 7 New York Regional Center for Diabetes Translation Research, Albert Einstein College of Medicine, Bronx, NY USA; 8 Ferkauf Graduate School of Psychology, Yeshiva University, Bronx, NY USA

**Keywords:** activity disruption, illness intrusiveness, life satisfaction, type 1 diabetes

## Abstract

Illness intrusiveness is a psychological process reflecting the perceived impact of illness-related disruptions on daily life in chronic conditions. Type 1 diabetes (T1D) disrupts daily activities through symptoms, self-care demands, and device use, yet little is known about how these disruptions shape psychosocial well-being. This study examined activity disruptions in adults with T1D using ecological momentary assessment (EMA) and tested whether illness intrusiveness mediates the relationship between activity disruptions and life satisfaction. A total of 196 adults with T1D completed 14 days of momentary activity disruption assessments and follow-up surveys assessing illness intrusiveness and life satisfaction. On average, participants reported disruptions in 11% of momentary daily activities, with notable variation across activity types, racial/ethnic groups, and treatment regimens. Activity disruption significantly predicted greater illness intrusiveness (β = 0.33, *p* < 0.001), which in turn predicted lower life satisfaction (β = -0.51, *p* < 0.001). Mediation analysis revealed that illness intrusiveness fully mediated the relationship when adjusted for income. Subtype analyses showed that device-related (indirect effect: β = -0.12, *p* < 0.001) and symptom-related disruptions (indirect effect: β = -0.12, *p* = 0.01) were fully mediated, whereas self-care disruptions were not associated with life satisfaction. Findings highlight the value of capturing real-time activity disruptions and suggest that illness intrusiveness may be a key explanatory mechanism linking activity disruption and psychosocial outcomes. Device burden and socioeconomic context emerged as factors shaping these relationships.

## Introduction

The disruption of one’s lifestyle is a common experience among individuals with chronic conditions (Alshammari et al., 2020; Estecha Querol et al., 2020), as the diagnosis and symptoms often abruptly alter routines and derail anticipated life trajectories (Bury, 1982; Kirk & Hinton, 2019). Illness-induced disruption can manifest in granular daily activities (activity disruption), psychological reflections (illness intrusiveness), and reduced overall well-being (life satisfaction). By understanding the relationships among these constructs, tailored psychoeducation and interventions can address the negative impacts of lifestyle disruption more efficiently (Martz et al., 2018; Roessler, 2004).

Illness intrusiveness offers a way to quantify illness-induced interference with valued activities, interests, and lifestyles in chronic conditions (Devins, 1994). Conceptually, it describes a psychological process reflecting how treatment demands and symptom burdens translate into perceived threats to well-being. Empirically, higher illness intrusiveness has been associated with poorer mental and physical health across chronic conditions, including end-stage renal disease, multiple sclerosis, and rheumatoid arthritis (Devins et al. 1993a, b; Gromisch et al. 2023).

While illness intrusiveness is a well-established predictor of well-being, less research has examined its antecedents. Recent conceptual work suggests that illness intrusiveness arises from experiences such as physical symptoms, experience of managing disease, and treatment access barriers (Do & Seo, 2022), indicating daily activity disruptions may serve as a precursor, by offering observable markers of these internalized burdens. For example, studies have shown that limited mobility and prolonged time on self-care routines are under-recognized disruptive experiences in daily activities that reduce life satisfaction and increase distress (Estecha Querol et al., 2020; Jowsey et al., 2014). These findings suggest that momentarily experienced disruptions in daily activities may represent an important, observable precursor to illness intrusiveness, with downstream effects on well-being outcomes such as life satisfaction.

Understanding the concrete, lived experience of illness-related disruptions could highlight opportunities to reduce these momentary disruptions, thus preventing the downstream effects on illness intrusiveness and life satisfaction. To capture those, ecological momentary assessment (EMA) provides a powerful methodological solution. EMA collects data multiple times a day in individuals’ natural environments, offering a fine-grained picture of daily activity and its interruptions (Hoppmann & Ho, 2015; Stone et al., 2007).

Type 1 diabetes (T1D) is recognized as among the most burdensome chronic conditions, as it intrudes on daily life through symptoms, self-care demands, and device use. T1D is characterized by a lifelong dependence on exogenous insulin, requiring intensive self-management to prevent acute and long-term complications (Livingstone et al., 2015). First and foremost, symptoms of T1D illustrate internal and external disruptions: concerns about glucose levels distract people’s attention, and symptomatic glycemic events hinder their physical functioning (Liakos et al., 2024). Moreover, to manage diabetes, individuals need to incorporate a range of self-management practices into daily activities, including diet, physical activity, insulin delivery, and blood glucose monitoring (Arafat et al., 2016). These demands require prolonged time and cognitive effort, with emerging evidence indicating that adults with T1D spend substantial portions of their day thinking about and managing their condition (Priesterroth et al., 2025). For instance, diabetes device use interrupts their working rhythm (Overgaard et al., 2020), and wearing devices leads to perceived and experienced negative social consequences (Tanenbaum & Commissariat, 2023). Thus, T1D is an opportune context for examining real-time disruption and its psychosocial impact.

Activity disruption may contribute to decreased life satisfaction by increasing illness intrusiveness. Decreased life satisfaction has been linked to the perceived impact and severity of diabetes (Stefanowicz-Bielska et al., 2025), and illness intrusiveness has been identified as a key predictor of life satisfaction in other chronic conditions (McFadden, 1995). However, no studies to our knowledge have investigated activity desruption in the context of the unique daily demands of type 1 diabetes management, or tested the hypothesis of relationships among activity disruption, illness intrusiveness, and life satisfaction. A more comprehensive understanding of how these disruptions affect psychological well-being is needed to guide future intervention efforts.

In adults with T1D, to address the gap, we analyzed a subset of data from an ecological momentary assessment (EMA) study to quantify real-time activity disruptions by symptom, device, and self-care in adults with T1D. Our first aim is to characterize activity disruptions in daily life of adults with T1D (e.g., what activities are disrupted, why, and how often; and among whom they are more common). Our second aim is to test whether illness intrusiveness mediates the relationship between activity disruption and life satisfaction. We hypothesize that the effect of externally observed activity disruptions on life satisfaction is mediated by internal perceptions of illness intrusiveness.

## Method

### Overview of study design

The data utilized in this study were derived from a larger project, Function and Emotion in Everyday Life with Type 1 Diabetes (FEEL-T1D); the full procedure and details are outlined in Pyatak et al. (2021). In this paper, we present a brief summary of methods pertaining to the current secondary analysis.

### Participants

Participants were recruited from 3 clinical sites in Los Angeles and New York City areas, which ensured the diversity of ethnicity and socioeconomic status of the participants. Adults aged 18–75 years with T1D for at least one year and on a stable diabetes regimen for over three months were included. Participants were required to have experience using a smartphone, sufficient manual dexterity and visual acuity, and no significant psychiatric (e.g., psychotic disorder, bipolar disorder, etc.) or cognitive impairments (e.g., intellectual disability, neurocognitive disorders, traumatic brain injury, etc.) that could interfere with study procedures. Individuals with planned medical procedures, recent infections, or adhesive allergies were excluded.

Researchers employed phone calls, mailings, email invitations, and health provider referrals to advertise the study and proceeded with participant recruitment. Study procedures were approved by the University of Southern California and the Albert Einstein College of Medicine institutional review boards. All participants were informed about the study aims, activities, benefits, and risks, and signed the consent form before engaging in study procedures. The recruitment for the original study was from June 2020 to February 2022, and no extra participants were enrolled after the closure.

### Procedure

#### Shipping of the study kit and training calls

To collect data remotely, we shipped each participant a kit containing two Abbott FreeStyle LibrePro Flash Glucose Monitoring System sensors (one primary and one backup), an Abbott CGM reader for sensor activation, a Xiaomi Mi A1 smartphone with all study apps preinstalled plus charging accessories, a detailed participant manual, and supplies to enhance device wearability and manage skin irritation (adhesive patches, barrier wipes, allergy‑relief spray, and hydrocortisone cream). A pre‑paid return package for shipping materials back after data collection was also included. Upon receiving their kit, participants joined a video‑conference training session with a research coordinator, who demonstrated sensor self‑application, proper operation of the CGM and smartphone, and all study procedures. CGM data from study-delivered devices were not visible to participants, while participants may continue using their own devices. Participants were required to demonstrate full understanding and ability to follow the protocol; those unable to do so were withdrawn from the study.

#### Data collection

Participants completed a baseline survey battery via REDCap (Harris et al., 2009) for demographic and clinical characteristics, and then 14 days of intensive longitudinal data collection using EMA surveys to capture activity disruption, study-delivered CGM to measure study-long glycemic variability. A follow-up evaluation was scheduled at the completion of EMA data collection, where participants completed follow-up surveys of life satisfaction and illness intrusiveness in RedCap, and were instructed to repack and return the study materials to the team.

#### Measures

*The 14-day EMA surveys* were implemented every 3 h for 5–6 times a day. Upon each prompt, surveys included approximately 30 items; more questions were added on the first morning and the final evening surveys. To assess activity disruption, participants were asked: (1) “What were you doing right before starting this survey?” with response options including work/school activities, traveling, relaxing/chilling, sleeping/napping, socializing, caring for myself, caring for others, doing housework/errands, fun/play/leisure activities, and other; these activity classifications were developed by occupational therapists based on the Occupational Therapy Practice Framework (American Occupational Therapy Association, 2020; Hernandez et al., 2021). The definitions and examples for each activity type were explained during baseline training and provided in a reference manual given to participants, but were not displayed alongside the response options during survey completion. Next, participants were asked (2) “Did your diabetes get in the way of doing this activity?”; and (3) If yes, they identified the reason(s) for disruption: symptoms, device, or self-care demands. The detailed questions and response options are shown in Supplemental Table 1. We calculated each participant’s activity disruption proportion by dividing the number of disrupted activities by the total number of activities reported over the course of the study, yielding a person-level metric of activity disruption.

*The study-provided continuous glucose monitoring (CGM) devices* (Abbott FreeStyle LibrePro) continuously recorded interstitial glucose levels throughout the 14-day data collection period. Glucose data were reprocessed using an algorithm equivalent to the Libre2 CGM, and used to derive a range of glycemic metrics for each participant, including the coefficient of variation (CV) to capture glycemic variability, glucose management indicator (GMI) as an estimate of overall glycemic quality, and percent time in clinically relevant glucose ranges, specifically, time in range (TIR; 70–140 mg/dL), and time spent in Level 1 and Level 2 hypoglycemia and hyperglycemia (Battelino et al., 2019).

*The Satisfaction with Life Scale (SWLS)* was applied to measure life satisfaction (Pavot et al., 1991). SWLS is a 5-item instrument with responses on the 7-point Likert scale (1 = “strongly disagree,” 7 = “strongly agree”). Total scores of life satisfaction range from 5 to 35 points, where higher scores suggest greater life satisfaction. The SWLS has shown reliability and validity to measure life satisfaction (Pavot et al., 1991) and is feasible for utility in T1D (Brzoza et al., 2022).

*The Adapted Illness Intrusiveness Rating Scale (AIIRS)* was used in this study to measure how T1D interferes with valued life domains (Devins, 2010). It covers five areas, including physical well-being and diet; work and finances; marital, sexual, and family routines; recreation and social relations; and other aspects of life. Each item was rated from 1 (“not very much”) to 7 (“very much”). Higher scores indicate greater perceived intrusiveness of the illness. In chronic condition samples, the AIIRS consistently demonstrates excellent internal reliability (α = 0.89).

#### Data analysis

Analyses were conducted in R (V4.5.0; R Core Team, 2025). Descriptive analyses were conducted to summarize participant demographics and clinical characteristics, quantify activity disruption (activity types, causes of interruption, and overall disruption proportion), and report psychosocial outcomes (AIIRS and SWLS scores).

Inferential analyses were performed. First, mixed effects logistic regression models were used to examine group differences in the activity disruption proportion across demographic and clinical variables. Second, we conducted linear regression models to test the associations among variables of activity disruption proportion, life satisfaction, and illness intrusiveness. The models were adjusted for potential demographic covariates (e.g., gender, age, race/ethnicity, education, income, and treatment regimens). For the last step, path analysis was conducted using the lavaan package (Rosseel, 2012) to test the hypothesis that illness intrusiveness mediates the relationship between activity disruption and life satisfaction. All regression and mediation models were adjusted for relevant demographic and clinical covariates (e.g., income, education, gender, BMI, age, TIR, and CGM treatment regimen), and statistical significance was defined as *p* < 0.05. We followed guidelines proposed by Rucker et al. (2011) and Hayes and Rockwood (2017) for mediation evaluation - mediation was considered partial if the indirect effect was statistically significant and the direct effect remained significant after including the mediator. It was considered full mediation if the indirect effect was significant and the direct effect became non-significant.

A full information maximum likelihood (FIML) estimator was used to account for missing data. FIML estimates model parameters directly from all available observed data by maximizing the likelihood of the observed data. This approach yields unbiased estimates under the assumption that data are missing at random (MAR; Cham et al., 2017).

## Results

Of the 207 participants who completed baseline data collection, 196 initiated and completed the EMA surveys and were included in the analyses. Eleven participants were excluded because they didn’t start EMA surveys or withdrew at the start of the EMA period; demographic characteristics of these participants, as compared to those included in the analyses, are presented in Supplemental Table 2. Compared with participants included in the analyses, excluded participants were significantly older (53.8 ± 13.4 years old vs. 39.6 ± 14.3 years old, *p* = 0.001), but did not differ significantly on other baseline demographic or clinical characteristics. Among the 196 participants who completed the EMA protocol, 193 completed the follow-up surveys. Their demographic information and clinical characteristics are presented in Table 1. The majority of participants identified as male (54.5%), Hispanic (40.8%), with a bachelor’s degree (28.1%), and with a mean age of 39.6 (SD = 14.3, range = 18 ~ 75) A significant portion of participants were using a continuous glucose monitoring (CGM) system (41.3%), while 23.0% were utilizing an automated insulin delivery (AID) system, in which the insulin pump makes automatic dosing adjustments based on input from a continuous glucose monitor (Templer, 2022).

**Table 1:** Baseline demographic and clinical characteristics of the participants (*N* = 196).

Demographic Characteristics	*n*	%	M	SD	Range
Age	196		39.55	14.30	18–75
Diagnosis time	193		20.68	12.56	1–57
Gender					
Male	107	54.6			
Female	89	45.5			
Language					
English	175	89.3			
Spanish	21	10.7			
Education					
Bachelor’s degree (BS/BA/AB)	55	28.1			
Some college but no degree	48	24.5			
High school graduate/diploma/GED	33	16.8			
Graduate degree	22	11.2			
Associate degree (AA)	18	9.2			
Some high school but no diploma	14	7.1			
8th grade or lower	3	1.5			
Race					
Hispanic	80	40.8			
Non-Hispanic White	56	28.6			
Non-Hispanic Black	29	14.8			
Multi-ethnicity	14	7.1			
Asian	7	3.6			
Other	6	3.1			
Employment					
Working now full-time	69	35.2			
Unemployed and looking for work	27	13.8			
Working now part-time	23	11.7			
Disabled	23	11.7			
Student	18	9.2			
Retired	15	7.7			
Full-time homemaker	9	4.6			
Other	8	4.1			
Annual household income					
Less than $25,000	47	24.0			
$25,000 to less than $34,999	29	14.8			
$35,000 to less than $49,999	14	7.1			
$50,000 to less than $74,999	15	7.7			
$75,000 to less than $99,999	14	7.1			
$100,000 to less than $199,999	17	8.7			
$200,000 or more	9	4.6			
Screen Site					
New York	79	40.3			
East Los Angeles	75	38.3			
West Los Angeles	42	21.4			
Clinical Characteristics	*n*	*%*	*M*	*SD*	*Range*
Number of CGM observations	159		1288.40	103.36	790–947
Glucose management indicator (GMI)^a^	159		7.70	1.30	5.67–13.35
Percent time in range (TIR)	159		53.51	22.01	4.24–97.79
Percent time < 54 mg/dL (very low)	159		0.67	1.46	0-11.79
Percent time 54–69 mg/dL (low)	159		3.61	3.97	0-22.45
Percent time 181–250 mg/dL (high)	159		21.97	9.65	0.15–52.32
Percent time > 250 mg/dL (very high)	159		20.24	20.32	0-89.76
Treatment Regimens					
AID	45	23.0			
Non AID CGM	70	35.7			
No CGM	81	41.3			
Activity Disruption Proportion	196		11.41%	16.31%	0-98.55%
Illness Intrusiveness (scoring range 1–7)	193		3.02	1.30	1-6.47
Life Satisfaction (scoring range 5–35)	193		21.97	7.47	5–35

^a^Glucose management indicator % = 3.31 + 0.02392 × mean glucose in mg/dL

### Activity disruption characteristics (*N* = 196)

14,495 EMA surveys were completed (Table 2). Each participant completed an average of 73.96 EMA surveys (SD = 15.81; range = 13–104). At the individual participant level, the average proportion of disrupted activities is 11.4% (SD = 16.3%), ranging from 0% to 98.55%. At the activity level, 11.6% were disrupted among all reported activities. Disruption proportions differed significantly across activity types (*p* < 0.001), and the post-hoc examination of standardized residuals indicated that relaxing/chilling activities were disrupted less often than expected, whereas self-care, sleeping/napping, and work/school activities were disrupted significantly more often than expected (|standardized residual| ≥2). The most commonly disrupted activities were caring for myself (15.1%), followed by caring for others (13.8%), and sleeping and napping (13.8%). Daily activities were most commonly disrupted by symptoms (54.6%), followed by self-care (36.1%), and devices (26.8%).

**Table 2:** Frequency and proportion of activity disruption, overall and across subtypes (symptom, device, self-care)

Activity types^a^	Reported	Disrupted		Symptom-caused^b^		Device-caused^b^		Self-care-caused^b^	
	*n*	*n*	%	*n*	%	*n*	%	*n*	%
Caring for myself	1,273	192	15.1	76	39.6	50	26.0	110	57.3
Sleeping/napping	1,940	268	13.8	162	60.4	100	37.3	73	27.2
Caring for others	383	53	13.8	41	77.4	14	26.4	11	20.8
Housework/errands	1,697	221	13.0	121	54.8	52	23.5	77	34.8
Fun/play/leisure	1,025	126	12.3	72	57.1	37	29.4	41	32.5
Other	615	71	11.5	38	53.5	16	22.5	34	47.9
Traveling	638	72	11.3	27	37.5	28	38.9	25	34.7
Work/school	2,775	290	10.5	180	62.1	58	20.0	81	27.9
Socializing	695	73	10.5	26	35.6	25	34.2	34	46.6
Relaxing/chilling	3,454	310	9.0	173	55.8	69	22.3	120	38.7
Total	14,496	1,677	11.6	916	54.6	449	26.8	606	36.1

*N* = 196

^*a*^*Disruption* proportion *differed significantly across activity types*,* χ² (9*, *N* = 14,*495) = 65.46*, *p* < 0.001

^*b*^ *Can select more than one/not mutually exclusive*

Frequency of reporting activity disruption did not differ significantly by age, gender, employment status, diabetes duration, or study-long glucose variability (coefficient of variation, CV), but it did differ significantly by race/ethnicity and treatment regimen (as shown in Table 3). Participants who identified as other race/ethnicity (including Asian, Native Hawaiian, and American Indian/Alaska Native; *n* = 27) had the highest proportion of activities disrupted by diabetes (0.09, 95% CI = 0.05 to 0.17), followed by non-Hispanic white (0.08, 95% CI = 0.05 to 0.12). Regarding treatment regimen, the group difference in the proportion of activity disruption was also significant, where participants on CGM (without automated insulin delivery) experienced the most activity disruption (0.08, 95%CI = 0.06 to 0.12) (Table 4).

**Table 3:** Group difference of activity disruption proportion across race/ethnicity and treatment regimen

Factors	Average proportion of activity disruption	SE	95% CI	*p*
Race/ethnicity				
Non-Hispanic White (*n* = 56)	0.08	0.02	0.05 to 0.12	0.03
Hispanic (*n* = 80)	0.04	0.01	0.02 to 0.05	
Non-Hispanic Black (*n* = 29)	0.05	0.02	0.02 to 0.09	
Other race/ethnicity (*n* = 27)	0.09	0.03	0.05 to 0.17	
Treatment regimen				
AID (*n* = 45)	0.06	0.02	0.04 to 0.1	< 0.01
Non-AID CGM (*n* = 70)	0.08	0.02	0.06 to 0.12	
No CGM (*n =* 81)	0.03	0.01	0.02 to 0.05	

*N* = 196

**Table 4:** AIIRS and SWLS scores, and association with activity disruption proportion

Model	Std.all	B	95%CI	*p*-value	*R* ^2^
SWLS ~ activity disruption	-0.01	-0.30	-6.80 to 6.20	0.93	0
SWLS ~ activity disruption (adjusted^a^)	-0.03	-1.18	-9.16 to 6.80	0.77	0.25
AIIRS ~ activity disruption	0.34	2.69	1.62 to 3.75	< 0.001	0.12
AIIRS ~ activity disruption (adjusted)	0.39	3.07	1.73 to 4.41	< 0.001	0.28
SWLS ~ AIIRS	-0.45	-2.60	-3.33 to -1.87	< 0.001	0.21
SWLS ~ AIIRS (adjusted)	-0.30	-1.74	-2.66 to -0.83	< 0.001	0.32

*N* = 193

^a^The adjusted covariates are gender, age, race/ethnicity, and treatment regimen

Exploratory stratified results of activity-level analyses by race/ethnicity and therapy regimen across activity types are provided in Supplemental Table 3. Among other race/ethnicity groups, work/school activities accounted for the largest proportion of disrupted activities (30.7%), whereas caring for others was the most frequently disrupted activity among Hispanic (14.3%) and Black (18.9%) participants. In contrast, White participants most frequently reported disruption during caring for myself (16.1%). With respect to treatment regimens, overall disruption was highest in CGM users who did not use AID (15.8%), moderate in AID users (10.8%) and lowest in participants who did not use CGM (7.9%), and device-caused disruption was substantially observed in participants using AID and non-AID CGM, whereas self-care-related disruptions were evident across all groups.

### Associations between activity disruption and psychosocial variables (*N* = 193)

Descriptive characteristics of AIIRS and SWLS are presented in Table 1. The linear regression analyses suggested that more activity disruptions are significantly associated with a higher level of illness intrusiveness (B = 3.07, 95%CI = 1.73 to 4.41). In turn, higher illness intrusiveness was significantly correlated with lower life satisfaction (B = -1.74, 95%CI = -2.66 to -0.83). In contrast, activity disruption did not directly predict life satisfaction (B = -1.18, 95%CI = -9.16 to 6.80). To further explore whether these associations varied by activity type, we conducted exploratory activity-specific regression analyses. As shown in Supplemental Table 4, activity disruptions across most activity types were positively associated with illness intrusiveness, whereas associations with life satisfaction were generally weaker and non-significant (ps = 0.07–0.56), indicating a pattern consistent with the primary association analyses.

### Mediation hypothesis tests

**Fig. 1:**
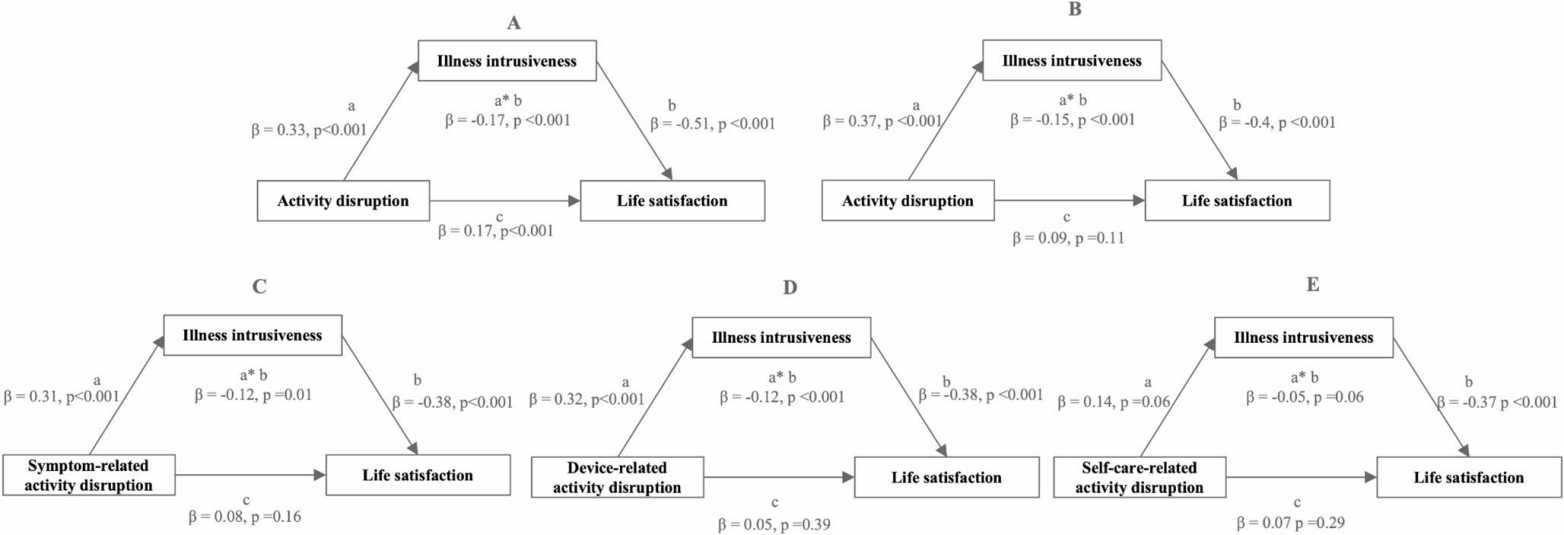
Panel of mediation models. a = effect of activity disruption on illness intrusiveness, b = effect of illness intrusiveness on life satisfaction, c = direct effect of activity disruption on life satisfaction, a*b = indirect effect of activity disruption on life satisfaction. Models A ~ E indicate different versions of mediation analysis: A = unadjusted original model, B = model with income adjusted, C = symptom-caused activity disruption with income adjusted, D = device-caused activity disruption with income adjusted, E = self-care activity disruption with income adjusted

The original mediation model (Fig. 1.A.), where activity disruption serves as a predictor, life satisfaction as an outcome, and illness intrusiveness as a mediator, indicated that activity disruption significantly predicted greater illness intrusiveness (path a: B = 2.52, 95% CI = 1.49 to 3.61), and illness intrusiveness, in turn, significantly predicted lower life satisfaction (path b: B = -2.92, 95%CI = -3.57 to -2.18). The indirect effect was significant (a*b: B = -7.35, 95% CI = -11.51 to -4.23). When illness intrusiveness was included in the model, the direct association between activity disruption and life satisfaction was also significant (path c: B = 7.23, 95% CI = 2.64 to 12.39), indicating partial mediation.

We adjusted the original mediation model for demographic variables, including income, education, gender, BMI, and age. While the significance of the indirect paths (a, b, and a*b) remained consistent with the original model, the direct effect of activity disruption on life satisfaction became non-significant (path c: B = 3.08, 95% CI = -2.06 to 8.35), suggesting a shift from partial to full mediation. To identify which demographic factor accounted for this change, we tested each covariate individually. The results indicated that income primarily contributed to the shift (Fig. 1.B.): when adjusting for income alone, the direct effect of activity disruption on life satisfaction also became non-significant (path c: B = 4.01, 95% CI = -0.95 to 8.93).

Further, the income-adjusted mediation model with illness intrusiveness as the mediator was tested separately for three subtypes of activity disruption: device-caused, symptom-caused, and self-care-caused (Fig. 1C, D, and E.). Full mediation was observed for both device- and symptom-related disruptions, where illness intrusiveness significantly explained the link between disruption and life satisfaction. In contrast, for self-care–related disruption, neither the direct nor indirect paths were significant, suggesting that illness intrusiveness does not mediate its relationship with life satisfaction.

## Discussion

This study provides one of the first real-time assessments of how T1D disrupts daily activities and how activity disruptions relate to psychosocial well-being. We found that activity disruption was common in adults with T1D and varied by race/ethnicity and treatment regimen. Notably, while disruption was not directly associated with life satisfaction, it significantly predicted greater illness intrusiveness, which in turn predicted lower life satisfaction, supporting a mediated path. The mediation patterns vary when adding demographic covariates and specifying subtypes of activity disruption.

The observed activity disruption patterns provide meaningful insight into how T1D interferes with everyday life. Consistent with existing literature, activities of caring for myself emerged as the most commonly disrupted, highlighting the persistent burden of diabetes management (Ahmad et al., 2023). Importantly, the wide variation in the proportion of disrupted activities, ranging from none to nearly all reported activities, suggests that the experience of activity disruption is highly individualized. This heterogeneity in the frequency of activity disruption highlights the need for precision monitoring (Hermanns et al., 2022) that extends beyond glycemic data to include broader psychological, contextual, and behavioral influences. By tailoring intervention strategies according to the diverse profiles of daily activities, support for reducing the burden of diabetes care can be improved.

Variation in activity disruption across race/ethnicity and treatment regimens further underscores the importance of tailoring the psychosocial intervention to individuals’ lived contexts. Differences in the types of activities most frequently disrupted across race/ethnicity groups may reflect variation in how disruption is experienced and interpreted, and they point to the value of developing more culturally responsive research and interventions. Regarding treatment regimens, the distinct patterns of device-related disruption among technology users, with the most advanced (AID) systems causing more disruptions than no technology but fewer than non-AID CGM, suggests that advances in diabetes technology may reshape, rather than eliminate, the ways daily activities are disrupted, highlighting the psychosocial dimensions of adopting and relying on diabetes technologies.

Although activity disruption was not significantly associated with life satisfaction in the linear regression analysis, the mediation model revealed a more complex pattern of associations. Contemporary mediation frameworks recognize that significant indirect effects may emerge even in the absence of a significant total effect, particularly when opposing mechanisms operate simultaneously (Bollen, 1989; Hayes & Rockwood, 2017). In our study, activity disruption was indirectly associated with lower life satisfaction through increased illness intrusiveness, while the direct association followed in the opposite direction, forming a competing mechanism presented as a non-significant correlation. This pattern suggests that examining mechanistic pathways offers additional insight beyond bivariate associations and provides important context for interpreting the role of activity disruption in daily life with T1D.

In the non-adjusted model, illness intrusiveness partially mediated the relationship between activity disruption and life satisfaction, aligning with our hypothesis grounded in both conceptual and empirical foundations (Alshammari et al. 2020; Devins et al. 1993a, b). We also interestingly observed a positive direct effect of activity disruption on life satisfaction, which invites reconsideration of how we operationalize disruption in chronic conditions. Traditionally, illness-induced disruptions are viewed as negative, often linked to compromised identity, strained management efforts, and diminished participation in social life (Estecha Querol et al., 2020; Potter, 2024). However, our findings suggest a more nuanced dynamic. Rather than viewing disruptions solely as obstacles, they may also serve as opportunities for reflection and adaptation. As Charmaz (1991) posits, individuals with chronic conditions often begin by interpreting disruption through the lens of acute illness, expecting temporary setbacks and a return to ‘normal.’ As disruptions recur and are embedded in daily life, they gradually shift the mindset to believe the condition is not an external event to overcome, but a part of the self (van Houtum et al., 2015). Although the positive direct effect became non-significant after adjusting for demographic characteristics, our finding still implies the future exploration of how activity disruption and illness intrusiveness interact with illness identity and self-management engagement.

When adjusting for income, the model changed from partial mediation to full mediation, meaning the previous significant direct effect of activity disruption on life satisfaction became non-significant. This pattern suggests that activity disruption alone does not directly determine life satisfaction, considering lived contexts; rather, its impact operates through illness intrusiveness. In this sense, activity disruption reflects momentary challenges encountered in daily life, whereas illness intrusiveness represents a psychological interpretive process through which these disruptions are integrated into one’s broader sense of living with a chronic condition. The central explanatory role of illness intrusiveness, which was well-established in chronic illness literature and now further verified by our data in T1D, explains how the same activity disruption may have different implications for life satisfaction, depending on how it is perceived, interpreted, and accumulated over time. From a clinical standpoint, our findings add specificity to existing recommendations for psychological support in diabetes care. While psychosocial support is already emphasized in current standards of care (Young-Hyman et al., 2016; ADA Professional Practice Committee for Diabetes, 2025), most interventions in T1D focus on diabetes distress, coping skills, or self-management behaviors more broadly. In contrast, illness intrusiveness has been more explicitly addressed in disability rehabilitation (Roessler, 2004) and type 2 diabetes (Martz, 2017) through coping with illness-related interference. To date, there has been limited investigation of illness intrusiveness as a distinct intervention target in T1D. Our findings suggest a direction for combining approaches to address discrete disruptions in daily activities and to support how individuals make sense of and adapt to these experiences.

The shift in the income-adjusted model points to the influence of external structural resources, particularly income, on subjective well-being. Life satisfaction, a well-recognized psychosocial outcome in chronic conditions (Brown et al., 1981), has been shown to improve with higher income levels in T1D populations (Imayama et al., 2011). Prior research suggests that individuals with higher income tend to have better self-management skills, improved HbA1c levels (Rechenberg et al., 2016), and a reduced risk of severe complications such as diabetic ketoacidosis (Lindner et al., 2018), all factors associated with greater life satisfaction. Our finding that the direct relationship between activity disruption and life satisfaction becomes nonsignificant after controlling for income suggests that individuals with higher income may be more resilient to the disruptive nature of diabetes, either because they experience fewer consequences or because they are better equipped to manage them. This highlights the importance of considering socioeconomic context in future research and interventions aimed at improving psychosocial well-being for people with chronic conditions.

The inconsistent mediation patterns across the three subtypes of activity disruption suggest that not all forms of disruption carry the same psychological meaning or consequence for individuals with T1D. While device- and symptom-related disruptions were significantly mediated by illness intrusiveness, self-management–related disruptions showed no direct or indirect effects. This pattern aligns with Devins’ (1994) conceptualization of illness intrusiveness as operating through two pathways: reduced access to positive reinforcement and diminished personal control. Device- and symptom-related disruptions are often unpredictable and externally imposed, undermining individuals’ sense of autonomy and emphasizing the perception that diabetes interferes with valued life activities. In contrast, self-management disruptions, though burdensome, may be more readily reframed as necessary actions under personal control, thus less psychologically intrusive (Charmaz, 1991).

In particular, the full mediation of device-related disruptions underscores the psychological cost of technology use in T1D. While diabetes technologies such as insulin pumps and CGM offer management and lifestyle benefits (Gonder-Frederick et al., 2016; Lomax et al., 2024), their utility is constrained by socioeconomic disparities. Individuals with lower income face compounded challenges, not only in accessing and affording devices (Sheikh et al., 2018) but also in building the required skills for their effective use (Sequeira et al., 2013). Our study findings reinforce this complexity. The delivered CGM sensors serving as newly adopted devices may cause skin irritation and adhesive issues (Cameli et al., 2022) which can be potentially interpreted as a disruptive discomfort. Additionally, participants using CGM without automated insulin delivery reported the highest rates of activity disruption (0.08, 95% CI = 0.06 to 0.12). Although study-delivered CGM were only for glucose data collection and offered no real-time feedback, around 50% of the sample were also using their personal CGM that is providing glucose results and alarms. This suggests that access to glucose data alone, without incorporating insulin technology, may increase the burden rather than reduce it, aligning with our findings that participants with Non-AID CGM experienced the highest proportion of activity disruption. To improve well-being in diabetes care, particularly among lower-income individuals, interventions must address both the physical access to devices and their lived experience of using them.

The model with symptom-related disruption as a predictor also illustrated a full mediation pattern. As symptoms were the main reason for reported activity disruption in our sample (54.6%), this suggests that bodily symptoms may represent a defining component of activity disruption in T1D. Unlike device-related interruptions, symptoms are experienced more directly and momentarily, and they often serve as the primary bodily cues signaling glucose dysregulation (Liakos et al., 2024). Given their immediacy and commonly perceived link to acute risk or future complications, symptoms may be especially likely to be interpreted as a disruptive experience. Given the role of symptom-related disruption in the construct of activity disruption and the full mediation pattern, our finding underscores that symptoms do not uniformly undermine well-being by their occurrence alone, but rather through the cognitive and emotional processes by which they are interpreted and accumulated over time. Momentary symptoms such as hypoglycemia may evolve into anticipatory concerns (e.g., fear of hypoglycemia; Liakos et al., 2024) or heightened awareness of long-term complications (Taraban et al., 2022), increasing psychological burden and perceived interference with daily life. Accumulatively, these processes may translate the symptomatic experiences into chronic distress and a heightened sense of illness intrusiveness, thereby diminishing overall life satisfaction. Together, these findings highlight the importance of addressing not only symptom management but also the psychological meaning-making of the symptoms in living with diabetes.

Although the present study focused on adults with T1D, our findings speak more broadly to the nature of activity disruption and its implications for well-being. This work provides an initial template for examining how momentary disruptions accumulate into illness intrusiveness and shape life satisfaction, and future research should extend this approach across the full age range of individuals with T1D and examine a broader set of psychosocial outcomes. For example, exploratory analyses suggest that activity disruption may also be related to diabetes-related quality of life, diabetes distress, perceived stress, and mental health (see Supplemental Table 5), all of which reflect related but distinct dimensions of lived experience with T1D. While certain sources of disruption, such as diabetes technologies, are specific to T1D, the construct of disruption has long been recognized across chronic conditions, from immediate interference to longer-term intrusiveness. Our EMA-based, data-driven approach to measuring activity disruption and modeling its indirect effects through illness intrusiveness offers a framework adaptable to other chronic conditions.

Several limitations of the study should be acknowledged. While our sample was relatively sociodemographically diverse compared to many prior behavioral studies in T1D, thereby strengthening the relevance of these findings for populations that are often underrepresented in behavioral health research, a concomitant limitation is that older adults were more likely to withdraw from study procedures upon initiation than younger adults; thus, our findings may not generalize as readily to older adults. Second, we started from a non-significant correlation between interested variables, which is inconsistent with conventional procedures for mediation testing. However, the theoretical justification and contemporary statistical perspective (Bollen, 1989; Hayes & Rockwood, 2017) support testing mediation without requiring a significant direct effect. Nevertheless, future research should explore additional moderators or mediators to fully explain the relationship. The third limitation emerges from the EMA study design. We aggregated activity disruption into a between-person proportion, which limited our ability to capture within-person variability, examine temporal sequencing, and potentially draw causal inferences. Future research should leverage the full granularity of EMA data to better understand dynamics in daily activities. Additionally, although EMA is widely regarded as a minimally disruptive method for capturing daily experiences, the repeated prompts of EMA may itself be perceived as intrusive for some participants, raising the possibility of conceptual overlap between survey intrusion and device-related disruption, particularly when data are collected via digital devices. While participants in this study were instructed to distinguish between device-related disruption and EMA completion, future research should more explicitly examine how individuals interpret EMA prompts in relation to perceived activity disruption. Moreover, rapid advances in diabetes technologies, particularly the increasing adoption of AID systems, may limit the generalizability of our findings. Our sample reflects a transitional period in technology use, and future research should examine how evolving devices reshape the experience of activity disruption. Finally, data collection occurred during COVID-19 (from June 2020 to February 2022), a period when public restrictions may have altered individuals’ routines and activity contexts. The pandemic impacts may limit the generalizability of our findings. Future research should replicate this work in post-pandemic settings to assess the external validity.

## Conclusion

Our study revealed the multifaceted nature of activity disruption in type 1 diabetes and proposed a mediation model in which illness intrusiveness mediates the impact of activity disruption on life satisfaction. We demonstrated that activity disruptions are common, yet highly individualized, and that their impact on life satisfaction is shaped by both internal perceptions and external resources, where illness intrusiveness serves as an explanatory mediator. The variability in mediation patterns across disruption subtypes further suggests the need for assessing detailed disruption experiences to address individualized challenges. Together, these insights advocate for a more nuanced and person-centered approach to unpack the complexity of daily activity disruptions in diabetes care.
